# Functional Assessment for Control of the Trunk Predicts Independent Walking in Patients with Stroke

**DOI:** 10.31662/jmaj.2024-0212

**Published:** 2024-11-01

**Authors:** Keisuke Sato, Takahiro Ogawa

**Affiliations:** 1 LIM Projects Inc., Nakagami, Japan; 2 Chuzan Hospital Clinical Education and Research Center, Okinawa, Japan; 3 Department of Rehabilitation Medicine, Aichi Medical University, Nagakute, Japan

**Keywords:** Cerebral Infarction, Trunk Function, Walking

## Abstract

**Introduction:**

This study examined the association of trunk function evaluated using Functional Assessment for Control of Trunk (FACT) with independent walking. It aimed to determine the effectiveness of the FACT cutoff score in predicting independent walking at hospital discharge.

**Methods:**

This retrospective observational study included patients with cerebral infarction. The patients were categorized into the independent (Functional Independence Measure [FIM] locomotion walking score of the patient was ≥6; n = 102) and dependent (≤5; n = 111) groups based on the FIM locomotion scale at discharge. Multivariate logistic regression analysis was employed to determine the significant independent variables on admission for predicting independent walking at discharge. Furthermore, the receiver operating characteristic was used to calculate the cutoff value for admission status.

**Results:**

A total of 213 patients (122 men and 91 women) were included in this study. The independent group had higher scores in FACT (15.0 [12.0-20.0] vs. 6.0 [2.0-12.0], *P* < 0.001) on admission than the dependent group. The results of the multivariate logistic regression analysis indicated that the factors associated with independent walking were the FACT and Mini-Mental State Examination-Japanese (MMSE-J) on admission. The optimal cutoff score for the FACT on admission was 8, and the area under the curve for the FACT scores on admission when discriminating between independent walking at discharge was 0.82.

**Conclusions:**

The results of this study can facilitate the optimization of patient rehabilitation as early as possible. The effects of improved trunk function require further validation through prospective observational studies.

## Introduction

After stroke, one of the goals of rehabilitation for patients is to gain the ability to walk independently. Loss of walking ability is recognized as the most disabling consequence of stroke ^(1)^. The ability to walk is crucial for patients with stroke as it is an important factor in determining independence in activities of daily living (ADL) ^(2), (3)^.

Understanding the factors associated with the acquisition of independent walking is necessary for the implementation of rehabilitation. Previous studies have reported several factors associated with walking ability in patients with stroke, including age, lower-limb muscle strength, cognitive function, and the ability to maintain a sitting position ^(4), (5)^. Sitting ability is reported to be a predictor of walking prognosis and is a highly credible method ^(6)^. Trunk function plays a pivotal role in the ability to resume a sitting position as it is required for a stable sitting and a standing position. The Functional Assessment for Control of Trunk (FACT) is one of the tools used to assess trunk function ^(7)^, particularly in patients with stroke who are unable to walk. Previous studies have demonstrated that the FACT score on admission can predict functional outcomes in patients with stroke ^(7)^. However, no studies have examined the association between FACT on admission and independent walking at discharge. Elucidation of this association can help improve the walking ability of patients with stroke. Hence, this novel study aimed to examine the association between FACT on admission and independent walking at discharge in post-stroke patients. The second aim was to explore the FACT cutoff score on admission to discriminate independent walking at discharge.

## Materials and Methods

### Ethical approval

This study was conducted in compliance with the principles of the Declaration of Helsinki and the ethical guidelines of Chuzan Hospital. The study protocol was approved by the Ethical Committee of Chuzan Hospital (approval number: 22-19). The requirement for informed consent was waived due to the retrospective nature of the study. The choice to opt out of the study was made through an announcement posted on the web page of the hospital. The patients were allowed to withdraw from the study at any time.

### Study design and patients

This retrospective observational study included patients with cerebral infarction and was conducted from June 2018 to October 2021. Patients who did not undergo evaluation for the severity of cerebral infarction, trunk function, cognitive function, or voluntary movements and those who were hospitalized for <14 days were excluded.

### Data collection

We extracted data on patient characteristics, including age, sex, type of stroke, history of stroke, history of fractures, days from onset to rehabilitation hospital admission, body mass index, Mini-Mental State Examination-Japanese (MMSE-J) score ^(8)^, Brunnstrom stage (BRS) ^(9)^, and FACT score, from the patients’ medical records ^(7)^. Stroke severity was assessed using the National Institutes of Health Stroke Scale (NIHSS) ^(10)^. ADL was assessed using the Functional Independence Measure (FIM) ^(11)^. Physical therapists and nurses with appropriate training assessed the NIHSS and FIM scores. The NIHSS defines a score of ≤5 points as minor and ≥6 points as mild ^(12)^, with a higher score indicating greater neurological severity.

### Independent variables

To identify the factors associated with independent walking in in-patient rehabilitation after stroke, we examined several independent variables representing cognitive function, motor development, and trunk function. In this study, the MMSE-J, BRS, and FACT were employed.

Cognitive function was assessed using the MMSE-J ^(8)^. An occupational therapist evaluated the MMSE-J score upon admission. The MMSE-J is used to screen for dementia ^(13)^ and consists of 11 items, with scores ranging from 0 to 30; higher scores indicate better cognitive function.

The BRS is used to evaluate motor development ^(9)^. In this study, it was evaluated upon admission by a physical therapist, with Stage 1 (flask, no voluntary movement) being the lowest and Stage 6 being the highest (isolated joint movement). In this study, we evaluated the lower-limb BRS of the patients.

Trunk function was assessed using the FACT ^(7)^ by a physical therapist. The FACT score is a valuable instrument for functional outcome prediction and enables a clear assessment of trunk function ^(7)^. The main categories for the FACT, which consists of 10 items, are the capacity to maintain a static sitting position (items 1 and 2) and the capacity to maintain a sitting position throughout upper- and lower-limb movements (items 3-10). For items 1-3, the scores range from 0 (unable) to 1 (able); for items 4-7, from 0 (unable) to 2 (able); and for items 8-10, from 0 (unable) to 3 (able). The FACT scores ranged from 0 to 20, with a higher score indicating better trunk function.

### Primary outcome

The primary outcome was whether the patients walked independently at discharge. If the FIM locomotion walking score of the patient was >6, the patient was deemed to be walking independently (i.e., modified independence with the device or complete independence). We divided the patients into the independent and dependent groups based on their FIM locomotion scale scores at discharge. Furthermore, we compared the patient characteristics between the groups.

### Statistical analysis

Mean ± standard deviation and median (interquartile range) were employed to define quantitative variables, including parametric and nonparametric ones. The normal distribution of the data was assessed using the Shapiro-Wilk test. The categorical variables are expressed as the number of patients (percentage). For the group comparisons, the chi-squared test was employed for categorical variables and the Mann-Whitney U test for quantitative variables. To compare factors on admission between the independent and dependent groups, univariate analysis was used. In addition to comparing the independent and dependent groups, the patients were categorized into the minor (≤5 points) and mild (≥6 points) groups based on their NIHSS score and by whether the patients had cardioembolic stroke. These additional comparisons were performed to compare the impact of cortical symptoms and neurological severity ^(14)^.

Multivariate logistic regression analysis was used to determine the significant independent variables on admission to predict independent walking at discharge. The covariates were adjusted for age, sex, NIHSS score on admission, MMSE-J score on admission, FACT score on admission, paralysis lower limbs BRS, and FIM locomotion walk on admission as independent variables. These variables are related to independent walking and walking ability ^(4), (5), (15)^.

Moreover, receiver operating characteristic (ROC) curves were used to plot sensitivity against 1- specificity. The area under the ROC curve (AUC) was calculated to determine whether the admission FACT had the ability to identify independent walking at discharge. Youden’s index was utilized to identify the optimal cutoff value for FACT on admission. Also, propensity score matching (PSM) was performed based on the calculated FACT cutoff values to match the background characteristics of the two groups. Logistic regression analysis was conducted using age, sex, NIHSS score on admission, MMSE-J score on admission, paralysis lower limbs BRS, and FIM locomotion walk on admission as explanatory variables. Matching was performed using propensity scores. The significance level was set at 5%. All statistical analyses were conducted using the R software (version 1.55) ^(16)^.

## Results

**Figure 1.**
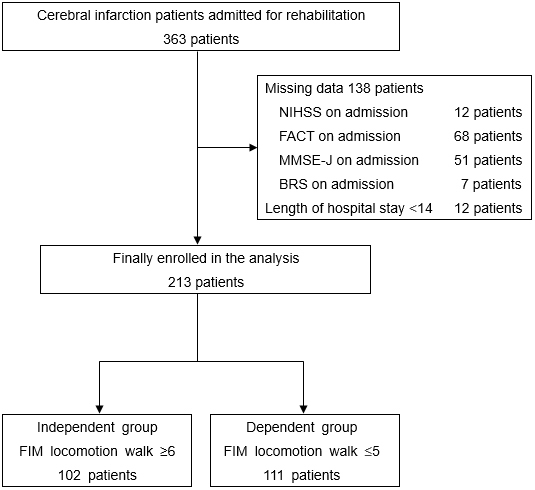
Inclusion diagram for the patients. NIHSS, National Institutes of Health Stroke Scale; FACT, Functional Assessment for Trunk Control; MMSE-J, Mini-Mental State Examination-Japanese; BRS, Brunnstrom stage; FIM, Functional Independence Measure.

During the study period, 363 patients with stroke were admitted to the hospital. We excluded 138 patients due to missing data and 12 with hospital stays of <14 days. Ultimately, 213 patients were included in the final analysis (Figure 1).

The baseline characteristics on admission for all patients and those grouped by NIHSS scores are presented in Table 1. All patients were categorized into the independent (n = 102, 47.9%) and dependent (n = 111, 52.1%) groups according to the FIM locomotion walking scores of ≥6 and <5, respectively (122 men and 91 women). The independent group was significantly younger than the dependent group (73.0 [65.3-83.0] vs. 82.0 [76.0-87.0], *P <* 0.001). On admission, the independent group had lower NIHSS (2 [1-5] vs. 7 [4-12], *P <* 0.001), higher FIM (75.5 [67.3-87.0] vs. 56.0 [37.0-66.0], *P <* 0.001), higher MMSE-J (24.0 [21.0-27.0] vs. 17.0 [11.0-22.0], *P <* 0.001), and higher FACT (15.0 [12.0-20.0] vs. 6.0 [2.0-12.0], *P <* 0.001) scores than the dependent group. Comparison by NIHSS severity showed significant differences in age, NIHSS, MMSE-J, FACT, and FIM between the groups. The baseline characteristics on admission of all patients and those grouped by the presence or absence of cardioembolic stroke are presented in Table 2. Comparison by the presence or absence of cardioembolic stroke showed significant differences in the NIHSS, MMSE-J, FACT, and FIM scores between the groups.

**Table 1.** Patient Characteristics on Admission to Rehabilitation Hospital (Comparison of all Participants and Grouping by NIHSS).

	Overall (n = 213)			NIHSS ≤ 5 points (n = 126)			NIHSS ≥ 6 points (n = 87)		
	Independent	Dependent	*P*-value	Independent	Dependent	*P*-value	Independent	Dependent	*P*-value
	(n = 102)	(n = 111)		(n = 81)	(n = 45)		(n = 21)	(n = 66)	
Age (years)	73.0 [65.3-83.0]	82.0 [76.0-87.0]	<0.001	75.0 [67.0-84.0]	81.0 [76.0-86.0]	0.024	69.0 [62.0-80.0]	82.0 [76.3-87.8]	<0.001
Sex, n (%)			0.019			0.131			0.130
Men	67 (65.7)	55 (49.5)		52 (64.2)	22 (48.9)		15 (71.4)	33 (50.0)	
Women	35 (34.3)	56 (50.5)		29 (35.8)	23 (51.1)		6 (28.6)	33 (50.0)	
Type of stroke, n (%)			0.286			0.745			0.210
Lacunar infarction	36 (35.3)	39 (35.1)		27 (33.3)	18 (40.0)		9 (42.9)	21 (31.8)	
Atherothrombotic cerebral infarction	56 (54.9)	53 (47.7)		45 (55.6)	22 (48.9)		11 (52.4)	31 (47.0)	
Cardiogenic cerebral embolism	10 (9.8)	19 (17.1)		9 (11.1)	5 (11.1)		1 (4.8)	14 (21.2)	
History of stroke, n (%)	27 (26.5)	38 (34.2)	0.236	24 (29.6)	14 (31.1)	1.000	3 (14.3)	24 (36.4)	0.064
History of fracture, n (%)	6 (5.9)	21 (18.9)	0.006	4 (4.9)	7 (15.6)	0.054	2 (9.5)	14 (21.2)	0.337
Days from onset to rehabilitation hospital (d)	16.0 [12.0-24.0]	20.0 [13.5-26.5]	0.029	16.0 [11.0-23.0]	17.0 [13.0-24.0]	0.249	18.0 [14.0-24.0]	20.0 [15.0-27.8]	0.460
BMI (kg/m^2^)	24.0 [21.3-26.7]	23.2 [20.4-25.3]	0.067	24.0 [21.4-26.5]	23.8 [20.7-25.7]	0.294	24.1 [20.1-27.2]	23.1 [20.3-24.8]	0.331
NIHSS score (points)	2.0 [1.0-5.0]	7.0 [4.0-12.0]	<0.001	2.0 [1.0-3.0]	4.0 [2.0-4.0]	0.001	9.0 [7.0-11.0]	10.0 [8.0-14.0]	0.029
MMSE-J score (points)	24.0 [21.0-27.0]	17.0 [11.0-22.0]	<0.001	24.0 [21.0-26.0]	17.0 [12.0-22.0]	<0.001	21.0 [16.0-28.0]	17.0 [11.0-21.8]	0.012
Paralysis lower limbs BRS, n			0.001			0.039			0.458
Ⅰ/Ⅱ/Ⅲ/Ⅳ/Ⅴ/Ⅵ	2/5/4/6/24/61	8/15/9/14/28/37		1/1/0/5/19/55	1/2/4/2/13/23		1/4/4/1/5/6	7/13/5/12/15/14	
FACT score (points)	15.0 [12.0-20.0]	6.0 [2.0-12.0]	<0.001	17.0 [14.0-20.0]	10.0 [5.0-15.0]	<0.001	11.0 [8.0-14.0]	3 [1.0-10.0]	<0.001
FIM score (points)	75.5 [67.3-87.0]	56.0 [37.0-66.0]	<0.001	77.0 [69.0-89.0]	63.0 [57.0-74.0]	<0.001	68.0 [62.0-84.0]	42.5 [31.8-58.0]	<0.001
Motor	51.5 [47.0-61.0]	33.0 [20.0-45.0]	<0.001	54.0 [49.0-62.0]	42.0 [35.0-49.0]	<0.001	46.0 [39.0-51.0]	26.0 [18.0-35.5]	<0.001
Cognitive	23.5 [20.0-26.0]	20.0 [14.0-24.0]	<0.001	24.0 [19.0-26.0]	22.0 [19.0-24.0]	0.184	23.0 [21.0-28.0]	17.0 [12.0-21.0]	<0.001

NIHSS, National Institutes of Health Stroke Scale; BMI, body mass index; MMSE-J, Mini-Mental State Examination-Japanese; BRS, Brunnstrom stage; FACT, Functional Assessment for Control of Trunk; FIM, Functional Independence Measure Values are expressed as means (SD) or medians [25th, 75th percentiles]

**Table 2.** Patient Characteristics on Admission to Rehabilitation Hospital (Comparison by All Participants and Type of Stroke).

	Overall (n = 213)			Cardioembolic (n = 29)			Non-cardioembolic (n = 184)		
	Independent	Dependent	*P*-value	Independent	Dependent	*P*-value	Independent	Dependent	*P*-value
	(n = 102)	(n = 111)		(n = 10)	(n = 19)		(n = 92)	(n = 92)	
Age (years)	73.0 [65.3-83.0]	82.0 [76.0-87.0]	<0.001	76.0 [65.3-84.5]	79.0 [71.0-83.5]	0.597	73.0 [65.8-83.0]	83.0 [76.0-87.0]	<0.001
Sex, n (%)			0.019			1.000			0.017
Men	67 (65.7)	55 (49.5)		6 (60.0)	11 (57.9)		61 (66.3)	44 (47.8)
Women	35 (34.3)	56 (50.5)		4 (40.0)	8 (42.1)		31 (33.7)	48 (52.2)	
Type of stroke, n (%)			0.286						0.764
Lacunar infarction	36 (35.3)	39 (35.1)					36 (39.1)	39 (42.4)
Atherothrombotic cerebral infarction	56 (54.9)	53 (47.7)					56 (60.9)	53 (57.6)
Cardiogenic cerebral embolism	10 (9.8)	19 (17.1)							
History of stroke, n (%)	27 (26.5)	38 (34.2)	0.236	1 (10.0)	8 (42.1)	0.107	26 (28.3)	30 (32.6)	0.631
History of fracture, n (%)	6 (5.9)	21 (18.9)	0.006	0 (0)	6 (31.6)	0.068	6 (6.5)	15 (16.3)	0.062
Days from onset to rehabilitation hospital (d)	16.0 [12.0-24.0]	20.0 [13.5-26.5]	0.029	18.0 [13.3-27.5]	23.0 [14.5-29.5]	0.383	16.0 [12.0-24.0]	18.5 [13.0-26.0]	0.068
BMI (kg/m^2^)	24.0 [21.3-26.7]	23.2 [20.4-25.3]	0.067	23.8 [22.1-28.5]	23.6 [20.4-25.5]	0.358	24.1 [21.2-26.5]	23.2 [20.5-25.1]	0.093
NIHSS score (points)	2.0 [1.0-5.0]	7.0 [4.0-12.0]	<0.001	1.5 [1.0-3.5]	12.0 [5.5-15.0]	<0.001	2.0 [1.0-5.0]	6.5 [4-10.0]	<0.001
MMSE-J score (points)	24.0 [21.0-27.0]	17.0 [11.0-22.0]	<0.001	23.5 [22.3-26.0]	17.0 [12.5-22.0]	0.027	24.0 [20.0-27.0]	15.5 [10.8-21.3]	<0.001
Paralysis lower limbs BRS, n			0.001			0.269			0.003
Ⅰ/Ⅱ/Ⅲ/Ⅳ/Ⅴ/Ⅵ	2/5/4/6/24/61	8/15/9/14/28/37		1/0/0/0/1/8	1/4/0/3/2/9		1/5/4/6/23/53	7/11/9/11/26/28	
FACT score (points)	15.0 [12.0-20.0]	6.0 [2.0-12.0]	<0.001	15.0 [11.0-17.0]	6.0 [1.0-13.0]	0.005	15.5 [12.0-20.0]	6.0 [2.0-12.0]	<0.001
FIM score (points)	75.5 [67.3-87.0]	56.0 [37.0-66.0]	<0.001	74.5 [64.3-84.3]	51.0 [27.5-65.5]	0.001	75.5 [67.8-87.0]	56.5 [38.0-66.0]	<0.001
Motor	51.5 [47.0-61.0]	33.0 [20.0-45.0]	<0.001	50.0 [43.3-62.3]	33.0 [16.0-41.5]	0.002	51.5 [47.0-61.0]	33.0 [20.8-45.5]	<0.001
Cognitive	23.5 [20.0-26.0]	20.0 [14.0-24.0]	<0.001	23.0 [22.0-24.0]	16.0 [12.0-22.0]	0.029	23.5 [19.8-27.0]	20.0 [16.0-24.0]	<0.001

BMI, body mass index; NIHSS, National Institutes of Health Stroke Scale; MMSE-J, Mini-Mental State Examination-Japanese; BRS, Brunnstrom stage; FACT, Functional Assessment for Control of Trunk; FIM, Functional Independence Measure Values are expressed as means (SD) or medians [25th, 75th percentiles]

Table 3 presents the results of the multivariate logistic regression analysis. Multivariate logistic regression analysis revealed that the MMSE-J (odds ratio [OR], 1.140; 95% confidence interval [CI], 1.07-1.21; *P <* 0.001) and FACT (OR, 1.170; 95% CI, 1.08-1.27; *P <* 0.001) scores on admission were significant predictors of independent walking at discharge.

**Table 3.** Multivariate Logistic Regression Model to Predict Independent Walking at Discharge (All Participants).

	Odds ratio	95% CI	*P*-value
Age	0.980	0.94, 1.02	0.335
Men	1.800	0.86, 3.78	0.120
NIHSS on admission	0.930	0.85, 1.02	0.122
MMSE-J on admission	1.140	1.07, 1.21	<0.001
FACT on admission	1.170	1.08, 1.27	<0.001
Paralysis lower limbs BRS on admission	1.130	0.85, 1.50	0.408
FIM locomotion walk on admission	1.120	0.84, 1.50	0.449

CI, confidence interval; NIHSS, National Institutes of Health Stroke Scale; MMSE-J, Mini-Mental State Examination-Japanese; FACT, Functional Assessment for Control of Trunk; BRS, Brunnstrom stage; FIM, Functional Independence Measure

**Figure 2.**
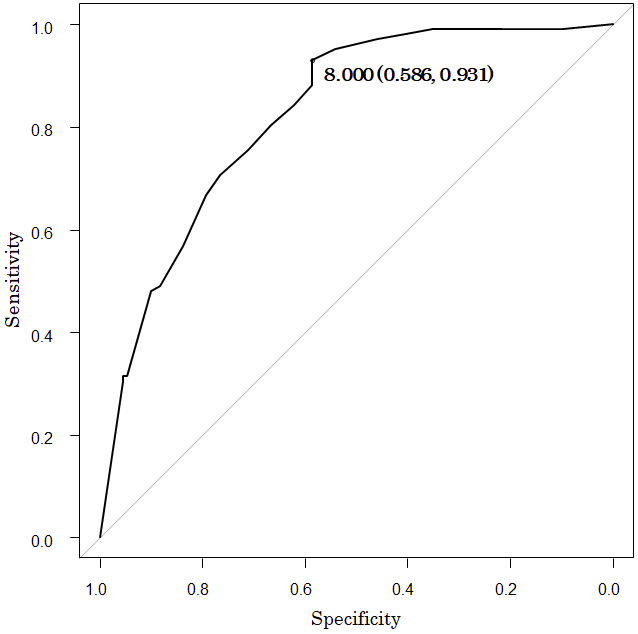
Receiver operating characteristic curve analysis of the Functional Assessment for Control of Trunk (FACT) score on admission for predicting independent walking at discharge (Functional Independence Measure locomotion walk ≥ 6). The optimal cutoff score for the FACT on admission was 8 (sensitivity, 93%; specificity, 59%).

Figure 2 presents the ROC curves of the FACT scores on admission in predicting independent walking at discharge. The optimal cutoff score for the FACT on admission was 8 (sensitivity 93%, specificity 59%), and the area under the ROC curve for the FACT score on admission was 0.82 (95% CI, 0.77-0.88).

Table 4 presents the patient characteristics before and after PSM based on the cutoff value (8) of FACT on admission. The group above the cutoff had a significantly higher rate of independent walking at discharge than the group below the cutoff (19 [45.2%] vs. 5 [11.9%], *P* = 0.001).

**Table 4.** Patient Characteristics before and after Propensity Score Matching (PSM) Based on the Cutoff Value (8 points) of FACT on Admission.

	Before PSM (n = 213)			After PSM (n = 84)		
	Group with scores above the cutoff	Group with scores below the cutoff	*P*-value	Group with scores above the cutoff	Group with scores below the cutoff	*P*-value
	(n = 141)	(n = 72)		(n = 42)	(n = 42)	
On admission						
Age (years)	77.0 [68.0-84.0]	83.0 [76.0-87.0]	<0.001	81.5 [73.3-86.8]	81.0 [70.3-85.0]	0.510
Sex: men/women, n (%)	84 (59.6)/57 (40.4)	38 (52.8)/34 (47.2)	0.381	24 (57.1)/18 (42.9)	21 (50.0)/21 (50.0)	0.662
Days from onset to rehabilitation hospital (d)	16.0 [12.0-24.0]	21.0 [15.0-27.0]	0.003	17.0 [13.0-26.0]	20.5 [15.0-25.5]	0.255
NIHSS score (points)	3 [1-6]	9 [5-14]	<0.001	7 [3.3-9]	6 [4-9.8]	0.829
MMSE-J score (points)	22.0 [16.0-26.0]	17.0 [9.8-22.0]	<0.001	17.5 [14.3-22.0]	20.5 [14.0-22.8]	0.460
Paralysis lower limbs BRS; Ⅰ/Ⅱ/Ⅲ/Ⅳ/Ⅴ/Ⅵ, n	2/9/6/10/34/80	8/11/7/10/18/18	<0.001	1/5/5/6/10/15	3/4/2/7/14/12	0.656
FIM locomotion walk score (points)	1.0 [1.0-4.0]	1.0 [1.0-1.0]	<0.001	1.0 [1.0-1.0]	1.0 [1.0-1.0]	0.726
At discharge						
Rehabilitation volume (min/d)	143.3 [130.0-156.6]	139.7 [125.4-150.8]	0.259	146.4 [136.3-162.2]	143.8 [129.3-153.6]	0.323
FIM locomotion walk score (points)	6.0 [5.0-7.0]	1.0 [1.0-5.0]	<0.001	5.5 [5.0-6.0]	4.0 [1.0-5.0]	<0.001
Independence (≥6), n (%)	95 (67.4)	7 (9.7)	<0.001	19 (45.2)	5 (11.9)	0.001

PSM, propensity score matching; BMI, body mass index; NIHSS, National Institutes of Health Stroke Scale; MMSE-J, Mini-Mental State Examination-Japanese; FACT, Functional Assessment for Control of Trunk; BRS, Brunnstrom stage; FIM, Functional Independence Measure Values are expressed as means (SD) or medians [25th, 75th percentiles]

## Discussion

This study demonstrates that higher FACT scores on admission are associated with independent walking at discharge. Furthermore, the results of the ROC curve analysis indicated that a cutoff score of 8 for the FACT on admission may predict independent walking at discharge in patients with stroke.

Patients with a higher FACT score on admission were more likely to walk independently. Verheyden et al. reported that trunk performance is significantly associated with walking ability ^(17)^. Studies have reported that good sitting position is one of the predictors of independent walking in patients with stroke ^(18)^ and that trunk function is essential for a good sitting posture. However, no studies have investigated independent walking as an outcome using FACT in patients with stroke. The FACT consists of items about movements commonly used in a clinical setting. The evaluation can be performed in a short period, with less physical burden on the subject. In this study, patients with higher trunk function assessed using the FACT on admission were more likely to walk independently at discharge.

The ROC curve regression resulted in a cutoff score of 8 for FACT. This score is highly sensitive, suggesting that a FACT score of <8 indicates that the patient cannot walk independently. Meanwhile, the specificity was lower than the sensitivity; hence, some subjects could not walk independently even with a FACT score of ≥8. The predictors of independent walking include not only trunk function but also factors such as intact corticospinal tracts, good leg strength, and lack of cognitive impairment ^(18)^. These findings indicate that in subjects with high trunk function, other factors may prevent them from walking independently. As for subjects with FACT scores of ≥8, rehabilitation focusing not only on trunk function but also on factors that interfere with independent walking is necessary. However, the AUC was as high as 0.82, suggesting that the FACT is a good predictor of walking prognosis. In this study, the FACT cutoff values on admission for predicting independent walking at discharge have several clinical implications. In patients with a FACT score of ≥8 or higher on admission, an intervention plan and discharge goals for the reacquisition of independent walking can be established early. However, for those with a FACT score of <8 on admission, more rehabilitation interventions are needed to regain the ability to walk independently. Karthikbabu et al. investigated the effects of trunk rehabilitation in patients with chronic stroke ^(19)^ and reported significant improvements in gait speed, cadence, and symmetry following trunk rehabilitation. In addition, Sorinola et al. evaluated the effectiveness of trunk training on functional outcomes ^(20)^. They reported that trunk training improved standing balance and mobility. Therefore, patients with a FACT score of <8 on admission have a better chance of achieving independent walking when provided with adequate rehabilitation aimed at improving trunk function. Early identification of patients who have difficulty walking independently may allow measures to be taken to help them acquire independent walking. Furthermore, it may lead to early optimization of patient care according to the results of the assessment at the time of admission ^(21)^.

This study has several limitations. First, a considerable number of individuals were excluded from the study due to missing data. Therefore, the results of this study cannot be generalized to patients with stroke. Second, the retrospective nature of the study may cause differences in the data regarding independent walking, such as the backgrounds of the patients.

### Conclusion

This study demonstrates that the FACT score on admission is a strong predictor of independent walking at discharge for patients with stroke. Patients with a FACT score of ≥ 8 are more likely to walk independently, which is useful for early rehabilitation planning. Contrarily, patients with a score of <8 require additional rehabilitation aimed at improving trunk function. This suggests that the FACT is an important tool to improve the walking prognosis of patients with stroke.

## Article Information

### Conflicts of Interest

None

### Acknowledgement

We would like to acknowledge all the patients who agreed to participate in this study.

### Author Contributions

KS and TO conceived and designed the study. KS performed the data collection and was supported by TO. KS conducted the data analysis with the support of TO. The initial drafts of the manuscript were written by KS and TO and critically reviewed and edited by the other authors. All authors agreed on the final version of the manuscript and are accountable for all aspects of the work.

### ORCID iD

Takahiro Ogawa: https://orcid.org/0000-0003-4775-5649

### Approval by Institutional Review Board (IRB)

None.
